# Diseases Linked to Polypharmacy in Elderly Patients

**DOI:** 10.1155/2017/4276047

**Published:** 2017-12-25

**Authors:** Ioannis Vrettos, Panagiota Voukelatou, Apostolos Katsoras, Despoina Theotoka, Andreas Kalliakmanis

**Affiliations:** 2nd Department of Internal Medicine, General and Oncological Hospital of Kifissia “Agioi Anargyroi”, Noufaron and Timiou Stavrou 14, Athens, Greece

## Abstract

**Introduction:**

Polypharmacy in several cases is deemed necessary and elderly patients are prone to this phenomenon. The objective of this study was to identify the prevalence and the predictors of polypharmacy among consecutively unplanned admissions of patients aged ≥65 years.

**Patients and Methods:**

In 310 patients (51% women), mean age 80.24 years (95% CI 79.35–81.10), demographic characteristics, medical history, medications, and cause of admission were recorded. Parametric tests and multiple logistic regression analysis were applied to identify the factors that have significant association with polypharmacy.

**Results:**

53.5% of patients belonged to polypharmacy group. In multivariate analysis the independent predictors of polypharmacy were arterial hypertension (*p* = 0.003, OR = 2.708, and 95% CI 1.400–5.238), coronary artery disease (*p* = 0.001, OR = 8.274, and 95% CI 3.161–21.656), heart failure (*p* = 0.030, OR = 4.042, and 95% CI 1.145–14.270), atrial fibrillation (*p* = 0.031, OR = 2.477, and 95% CI 1.086–5.648), diabetes mellitus (*p* = 0.010, OR = 2.390, and 95% CI 1.232–4.636), dementia (*p* = 0.001, OR = 4.637, and 95% CI 1.876–11.464), and COPD (*p* = 0.022, OR = 3.626, and 95% CI 1.208–10.891).

**Conclusions:**

Polypharmacy mainly was linked to cardiovascular diseases. If deprescribing is not feasible, physicians must oversee those patients in order to recognise early, possible drug reactions.

## 1. Background

Between the determinants of polypharmacy increased age was highlighted as a major one [1–4] as aging is characterized by the presence of multiple independent chronic diseases in the same person, a fact that is almost always accompanied by multiple drug use [5].

Factors that contribute to the development of polypharmacy are the lower thresholds for treating risk factors in preventative medicine, the new drug treatments that are now available for managing many chronic illnesses, and the new indications for older drug treatments [6].

In Greece, data concerning the factors that lead to multiple drug use in the elderly are scarce. The present study was undertaken to identify factors associated with polypharmacy among consecutively unplanned admissions of patients aged ≥65 years, in a department of internal medicine.

## 2. Patients and Methods

### 2.1. Study Population

A prospective study was conducted among patients older than 65 years, consecutively admitted through the emergency department of General and Oncological Hospital of Kifissia “Agioi Anargyroi” during March 2015 to December 2016. For each patient, a study physician completed a questionnaire at admission. Patients' demographic and social characteristics (age, gender, marital status, educational level, and living alone), medical history (comorbidities, physical activity status), and medication history (number and names of medications) were recorded by the study physician. Comorbidity was measured using the Charlson Comorbidity Index (CCI) [7] and physical activity status was evaluated using the Katz Index [8]. Information regarding demographic and social characteristics, medical history, and medication history was obtained by asking either the patients or their caregivers, when patients were not able to communicate. The research protocol was approved by the institutional ethical and scientific committee. Informed consent was obtained from participating patients or from their family members.

### 2.2. Definition of Polypharmacy

For this study polypharmacy was defined as the concurrent use of five or more medications.

### 2.3. Statistical Analysis

Categorical variables are expressed as frequencies and percentages. Continuous variables are expressed as means ± 95% confidence interval. The Kolmogorov–Smirnov test was used to assess the distribution of evaluated continuous variables. We found that age was normally distributed while the CCI had non-Gaussian distribution. For categorical variables, a chi-square test was applied to evaluate whether the prevalence of polypharmacy differed across patients' sociodemographic characteristics and across patients' medical and medication history. Student's *t*-test and Mann–Whitney *U* test were used to compare age and CCI, respectively, between polypharmacy and nonpolypharmacy group. Age was analyzed both as a continuous and as a categorical variable and categorized into two groups (65–80 and 81–100 years). Results were considered statistically significant when *p* < 0.05. Variables that had a significant influence on the presence of polypharmacy in the bivariate analysis were proceeding into the separate logistic regression analysis to identify the most important ones, associated with polypharmacy. By means of this method, the odds ratio (OR) and 95% confidence interval were designated, for the most important determinants of polypharmacy. All analyses were performed using SPSS v22.0.

## 3. Results

During the study period, 310 patients older than 65 years were admitted to the medical unit through the emergency department. Mean age was 80.24 years (95% CI 79.35–81.10, range 65–99). There were 158 women (51%) and 152 men (49%). 166 patients (53.5%) belonged to polypharmacy group. The mean number of medications was 7.32 (95% CI 6.99–7.63) in polypharmacy group and 2.63 (95% CI 2.40–2.85) in nonpolypharmacy group. The sociodemographic and medical history differences between patients in polypharmacy and in nonpolypharmacy group are presented in Tables 1 and 2, respectively.

Statistical difference was found when we compared the polypharmacy and the nonpolypharmacy groups according to CCI (*p* = 0.001, *U* = 7798.000), age group (*p* = 0.050, *χ*^2^ = 3.951), and the living status (*p* = 0.017, *χ*^2^ = 6.347). Diseases that differed statistically significantly between the two groups are presented in Table 2.

In multivariate analysis the independent predictors of polypharmacy were arterial hypertension (*p* = 0.003, OR = 2.708, and 95% CI 1.400–5.238), coronary artery disease (*p* = 0.001, OR = 8.274, and 95% CI 3.161–21.656), heart failure (*p* = 0.030, OR = 4.042, and 95% CI 1.145–14.270), atrial fibrillation (*p* = 0.031, OR = 2.477, and 95% CI 1.086–5.648), diabetes mellitus (*p* = 0.010, OR = 2.390, and 95% CI 1.232–4.636), dementia (*p* = 0.001, OR = 4.637, and 95% CI 1.876–11.464), and COPD (*p* = 0.022, OR = 3.626, and 95% CI 1.208–10.891).

## 4. Discussion

According to the results of the present study, among elderly patients needing hospitalization up to 50% received five or more medications. In those patients polypharmacy was mainly linked to cardiovascular diseases (coronary artery disease, atrial fibrillation, arterial hypertension, and heart failure), diabetes mellitus, chronic obstructive pulmonary disease (COPD), and dementia.

It is not surprising that polypharmacy was linked to cardiovascular diseases. Polypharmacy in cardiovascular diseases is guided by evidence based guidelines recommending treatment with multiple drug classes [9]. For example, in heart failure the progressive use of multiple drugs and a complex therapeutic regimen are common and are driven by international guidelines. Even more so, elderly patients with heart failure often have comorbidities that require additional specific treatment, thus increasing the number of medications [10]. In a study of patients with self-reported heart failure the proportion of patients with heart failure who had five or more comorbid chronic conditions and the mean number of prescription medications increased from 42.1% to 58.0% and from 4.1 to 6.4 prescriptions, respectively, over a twenty-year period [11].

Coronary artery disease [12, 13], atrial fibrillation [13], and hypertension [3, 14] have previously been reported as predictors of polypharmacy. Consumption of more than five drugs was reported by 71% of patients with coronary artery disease [15] and by 40% of patients with atrial fibrillation [16]. In hypertensive patients the concurrent use of different drug classes (adrenergic inhibitors, vasodilators, beta blockers, and diuretics) is common as the risk of cardiovascular mortality is high on this population and blood pressure control is important for long term survival [17].

In this study polypharmacy was also significantly associated with diabetes mellitus, a finding consistent with previous studies [13, 17, 18]. In diabetic patients, several factors contribute to polypharmacy. Both the importance for a tight glycaemic control and multiple comorbidities associated with diabetes mellitus require the use of multiple drugs [19]. Consequently, the high number of prescribed medications in patients with diabetes is compatible with guideline recommendations [20].

Likewise, international guidelines recommend the use of a variety of drugs for the treatment of COPD [21] and the number of respiratory drugs in COPD patients increases as the severity of symptoms increases [22].

The association between polypharmacy and dementia is known and seems to be bidirectional. According to Clague et al., patients with dementia have more comorbidities and consequently receive more medications than those without dementia [23]. From another point of view polypharmacy increases the risk of potential inappropriate medication use, and as some potential inappropriate medications may have cognition-impairing effects, prolonged multiple drug use may result in dementia [24].

Our study has some limitations. The major limitation is that the study design did not allow making general conclusions concerning the prevalence of polypharmacy in the whole community, since the sample originates from the hospital. Another limitation of our study was the fact that it was conducted in only one department of internal medicine of a tertiary care hospital. However, we believe that the patients profile did not differ from that of patients attending the emergency departments of other tertiary hospitals and so the sample is representative of this patient's population.

## 5. Conclusions

Despite the limitations the present study represents an attempt to understand the complicated phenomenon of polypharmacy. Elderly patients taking five or more medications suffer predominantly from cardiovascular diseases. Nevertheless, “polypharmacy is not synonymous with inappropriate treatment” [25] and maybe in several cases multiple drug use is necessary and appropriate. In these cases, polypharmacy is a problem we must learn to live with and instead of deprescribing physicians must oversee those patients in order to recognise early, possible drug reactions.

## Figures and Tables

**Table 1:** Comparison of sociodemographic characteristics between patients belonging to polypharmacy and nonpolypharmacy group.

Sociodemographic characteristics	Polypharmacy group *n* = 166 (53.5%)	Nonpolypharmacy group *n* = 144 (46.5%)	Statistical significance
Gender			
Males	76 (45.8%)	76 (52.8%)	NS
Females	90 (54.2%)	68 (47.2%)	
Age (95% CI) (years)	80.64 (79.39–81.88)	79.77 (78.48–81.00)	NS
Age group			
65–80 (years)	61 (36.7%)	69 (47.9%)	*p* = 0.050 (*χ*^2^ = 3.951)
81–99 (years)	105 (63.3%)	75 (52.1%)	
Marital status			
Married	79 (47.6%)	72 (50.0%)	NS
Unmarried	3 (1.8%)	4 (2.8%)	
Divorced	3 (1.8%)	5 (3.5%)	
Widowed	81 (48.8%)	63 (43.8%)	
Educational status			
Primary	125 (75.3%)	108 (75.5%)	NS
Secondary	31 (18.7%)	23 (16.1%)	
Technological Education Institution	3 (1.8%)	4 (2.8%)	
University	7 (4.2%)	8 (5.6%)	
*Missing*	0	1	
Living alone			
*Yes*	21 (12.7%)	34 (23.6%)	*p* = 0.017 (*χ*^2^ = 6.347)
*No*	145 (87.3%)	110 (76.4%)	
Katz index (95% CI)	4.05 (3.68–4.40)	4.17 (3.72–4.51)	NS

NS: nonsignificant; CI: confidence interval.

**Table 2:** Comparison of medical history between patients in polypharmacy and nonpolypharmacy group.

Medical history	Polypharmacy group *n* = 166 (53.5%)	Nonpolypharmacy group *n* = 144 (46.5%)	Statistical significance
*Morbidity*			
Arterial hypertension			
Yes	125 (75.3%)	79 (54.9%)	*p* = 0.001
No	41 (24.7%)	65 (45.1%)	(*χ*^2^ = 14.317)
Hyperlipidemia/dyslipidemia			
Yes	49 (29.5%)	20 (13.9%)	*p* = 0.001
No	117 (70.5%)	124 (86.1%)	(*χ*^2^ = 10.885)
Diabetes mellitus			
Yes	72 (43.4%)	30 (20.8%)	*p* = 0.001
No	94 (56.6%)	114 (79.2%)	(*χ*^2^ = 17.745)
Stroke			
Yes	21 (12.7%)	5 (3.5%)	*p* = 0.004
No	145 (87.3%)	139 (96.5%)	(*χ*^2^ = 8.454)
Heart failure			
Yes	31 (18.7%)	4 (2.8%)	*p* = 0.001
No	135 (81.3%)	140 (97.2%)	(*χ*^2^ = 19.456)
Atrial fibrillation			
Yes	54 (32.5%)	13 (9.0%)	*p* = 0.001
No	112 (67.5%)	131 (91.0%)	(*χ*^2^ = 25.140)
Coronary artery disease			
Yes	51 (30.7%)	7 (4.9%)	*p* = 0.001
No	115 (69.3%)	137 (95.1%)	(*χ*^2^ = 33.909)
Chronic renal failure			
Yes	9 (5.4%)	1 (0.7%)	*p* = 0.023
No	157 (94.6%)	143 (99.3%)	(*χ*^2^ = 5.520)
COPD			
Yes	23 (13.9%)	7 (4.9%)	*p* = 0.011
No	143 (86.1%)	137 (95.1%)	(*χ*^2^ = 7.137)
Dementia			
Yes	31 (18.7%)	11 (7.6%)	*p* = 0.005
No	135 (81.3%)	133 (92.4%)	(*χ*^2^ = 8.018)
Benign prostate hyperplasia			
Yes	14 (8.4%)	4 (2.8%)	*p* = 0.049
No	152 (91.6%)	140 (97.2%)	(*χ*^2^ = 4.510)
Thyroid diseases			
Yes	24 (14.5%)	9 (6.3%)	*p* = 0.026
No	142 (85.5%)	135 (93.8%)	(*χ*^2^ = 5.461)
Hyperuricemia			
Yes	19 (11.4%)	4 (2.8%)	*p* = 0.004
No	147 (88.6%)	140 (97.2%)	(*χ*^2^ = 8.435)

CCI (95% CI)	5.77 (5.54–5.99)	5.13 (4.79–5.50)	*p* = 0.001 (*U* = 7798.000)

COPD: chronic obstructive pulmonary disease; CCI: Charlson Comorbidity Index; CI: confidence interval.
